# Effect of Coconut Fiber Length and Content on Properties of High Strength Concrete

**DOI:** 10.3390/ma13051075

**Published:** 2020-02-28

**Authors:** Waqas Ahmad, Syed Hassan Farooq, Muhammad Usman, Mehran Khan, Ayaz Ahmad, Fahid Aslam, Rayed Al Yousef, Hisham Al Abduljabbar, Muhammad Sufian

**Affiliations:** 1 NUST Institute of Civil Engineering, National University of Sciences and Technology, Sector H-12, Islamabad 46000, Pakistan; syed2arqam@gmail.com (S.H.F.); concrete_157@yahoo.com (M.U.); 2 Department of Civil Engineering, Dalian University of Technology, Dalian 116024, China; 3 Department of Civil Engineering, COMSATS University Islamabad, Abbottabad Campus 22060, Pakistan; ayazahmad@cuiatd.edu.pk; 4 Department of Civil Engineering, College of Engineering in Al-Kharj, Prince Sattam bin Abdulaziz University, Al-Kharj 11942, Saudi Arabia; r.alyousef@psau.edu.sa (R.A.Y.); H.alabduljabbar@psau.edu.sa (H.A.A.); 5 Department of Civil Engineering, Southeast University, Nanjing 210096, China; engr.m.sufian91@gmail.com

**Keywords:** concrete, coconut fiber, mechanical properties, microstructure

## Abstract

Recently, the addition of natural fibers to high strength concrete (HSC) has been of great interest in the field of construction materials. Compared to artificial fibers, natural fibers are cheap and locally available. Among all natural fibers, coconut fibers have the greatest known toughness. In this work, the mechanical properties of coconut fiber reinforced high strength concrete (CFR-HSC) are explored. Silica fume (10% by mass) and super plasticizer (1% by mass) are also added to the CFR-HSC. The influence of 25 mm-, 50 mm-, and 75 mm-long coconut fibers and 0.5%, 1%, 1.5%, and 2% contents by mass is investigated. The microstructure of CFR-HSC is studied using scanning electron microscopy (SEM). The experimental results revealed that CFR-HSC has improved compressive, splitting-tensile, and flexural strengths, and energy absorption and toughness indices compared to HSC. The overall best results are obtained for the CFR-HSC having 50 mm long coconut fibers with 1.5% content by cement mass.

## 1. Introduction

### 1.1. High Strength Concrete

Concrete is a widely used construction material because it is economical, locally available, and widely applicable [1]. Nowadays, concrete with higher strength and toughness is a requirement of construction industry [2]. High strength concrete (HSC) has improved mechanical properties compared to normal strength concrete (NSC); however, it is more brittle [1]. In the civil engineering construction industry, HSC has a variety of applications. In high rise buildings, HSC decreases the dead load of structures and also avoids larger sized columns [3]. Also, HSC reduces the dead load of girders in long span bridges by reducing their section sizes, which ultimately reduces the size of piers [4]. HSC is more durable than NSC as it possesses high density and low permeability, which increases its resistance to deleterious effects [5,6]. With the evolution of the construction industry, there is a need for new types of concretes having improved properties such as high strength, energy absorption capacities, and ductility [7]. Examples of new types of concrete are HSC, fiber reinforced high strength concrete, and high-performance concrete (HPC). Such concretes show substantially improved properties over conventional concrete [8]. The factor which limits the utilization of HSC is its brittleness. Additionally, for the production of high strength concrete, supplementary cementitious materials like silica fume, blast-furnace slag, and fly ash are used as part of the binder [9]. The use of these materials as replacements for cement reduces the porosity of concrete in the long term [10]. Conversely, with the addition of these cementitious materials, the brittleness is increased [11]. Therefore, controlling the brittleness of HSC is an important aspect in concrete technology that needs to be investigated.

### 1.2. Natural Fibers in Normal Strength Concrete

The mechanical properties of concrete can be enhanced by using natural or artificial fibers [12,13,14]. Eswari et al. [15] reported that concrete strength, ductility, damage tolerance in flexure, and energy absorption capacity can be enhanced by the incorporation of fibers in the concrete. The addition of fibers delays crack propagation and improves the stress distribution in the matrix at the time of loading [16]. Nowadays, natural fibers are incorporated in concrete to produce materials with improved strength and toughness [17,18]. Natural fibers have been used by researchers as alternatives to synthetic fibers in composites like concrete [19,20,21]. Natural fibers are very cheap compared to synthetic fibers, and are locally available in many countries. Natural fibers include coconut, bamboo, jute, palm, sisal, hemp, banana, kenaf bast, pineapple leaf, flax, ramine bast, sugarcane, abaca leaf, and cotton fibers. The use of these fibers as a reinforcement can improve the properties of the composite at a relatively low cost. Compared to synthetic fibers like steel, natural fibers are flexible and easy to handle, especially when they are used in large quantities [22]. Coconut fibers have the highest toughness among all known natural fibers, and are capable of taking 4–6 times more strain than other fibers [22,23]. A study by Ali et al. [22] was based on the mechanical and dynamic properties of coconut fiber reinforced concrete (CFRC). The influence of variations in the length and content of coconut fibers was analyzed. The lengths of the fibers were 25, 50, and 75 mm and the percentages by mass were 1%, 2%, and 3%. Two types of mix design were adopted for two types of concrete for a comparison between plain concrete (PC) and CFRC. The mix design ratio for cement, sand, and aggregates of PC was 1, 2, and 2, respectively, with a water:cement (w:c) ratio of 0.48. The same mix design of PC was adopted for CFRC with an increased w:c ratio. The increment of water for the CFRC mix was done stepwise; this practice was adopted to avoid the bleeding of concrete. The w:c ratio of the CFRC mix varied from 0.49 to 0.62. It was also observed from our experimental work that the value of the water:cement ratio of all CFRCs was higher than that of PC. It was also noted that as the fiber content increased, the value of slump decreased, so the value of slump of all CFRCs was shown to be less than that of PC. The experimental test results indicated that concrete with coconut fibers has improved toughness and flexural strength. The compressive strength (σ), compressive toughness, modulus of rupture (MOR), and flexural total toughness index of CFRC were improved to 4%, 21%, 2%, and 910% with 50 mm long fibers length. The compressive strength, splitting tensile strength (STS), modulus of rupture, and toughness index for PC and CFRC reported in the study by Baruah and Talukdar [24] are shown in Table 1. The best overall performance was reported for CFRC with a 2% volume fraction.

Natural fibers like coconut husk, bamboo, sugarcane bagasse, sisal, jute, and wood in concrete composites have also been suggested as application materials for construction works [25]. Cook et al. [26] performed an experimental study on fiber reinforced cementitious composites with variations in fiber length and volume. The discussion was based on fiber reinforced cement composites as low cast materials specially for roofing. The material parameters were the fiber length and volume and the compacting and casting pressure. All testing in this experimental work was based on ASTM standards. Permeability, bending, impact, water absorption, and dimensional stability tests were performed for evaluation and comparison. Based on the experimental results from the performed tests, it was observed that a composite with fiber of 37.5 mm in length and a fiber volume of 7.5% was optimal. It was also revealed that the cost of the proposed composite was lower compared to most of the others composites like corrugated galvanized iron, asbestos-cement sheeting, and bagasse-thermoset composites. Li et al. [27] studied coir fibers of two different length (20 and 40 mm) for two types of composites; longer fibers were also treated with 1% NaOH solution. For the production of coir fiber reinforced cementitious composites (CFRCC), coir fibers were taken into consideration and combined with chemical agents (dispersant, defoamer, or wetting agent) and cementitious material. The toughness index, flexural stress, elastic stress, and toughness of CFRCC were measured. It was observed that the CFRCC samples were 5–12% lighter than conventional mortar, and that the flexural properties were enhanced. The result also indicated that the toughness properties and toughness index were enhanced by more than ten times. The scanning electron microscopy (SEM) microstructure images showed improved physicochemical bonding of the treated CFRCC.

### 1.3. Coconut Fiber with Mineral Admixture in Concrete

Khan and Ali [13] studied medium strength concrete (MSC) and medium strength coconut fiber reinforced concrete (MSCFRC) with a combination of optimized silica fume and different contents of super plasticizer, i.e., 0%, 0.5%, 1%, and 1.5% by mass. The mix design ratio of MSCFRC was 1:2:2 (cement:sand:aggregate) with a water:cement ratio of 0.5:1,respectively. A 15% silica fume content by mass was used for MSCFRC. Coconut fiber of 50 mm length and 2% content by mass was added. The strength, crack behavior, absorbed energy, and toughness indexes were discussed. The stress–strain curves, load–deflection curves, and load–time curves of all the MSC and MSCFRC specimens with different percentages of super plasticizer were noted. It was revealed that MSC_1_ and MSCFRC_1_ showed the best overall performance, indicating that the optimal super plasticizer content is 1% by mass. Thus, it was concluded that MSCFRC with an optimized silica fume (15%), coconut fiber (2%), and super plasticizer (1%) content can be used for civil engineering applications. An analysis of the mechanical properties of fly ash silica-fume plain concrete (FA-SPC) and fly ash silica-fume coconut fiber reinforced concrete (FA-SCFRC) was performed by Khan and Ali [28]. The percentages of the different materials were taken by cement mass, e.g., silica fume comprised 15% and fly ash 0%, 5%, 10%, and 15%. Two percent by mass of coconut fibers with a length of 50 mm was added. Stress–strain curves, load–deflection curves, and load–time curves for FA-SPC and FA-SCFRC were plotted in order to investigate the compression, flexure, and split-tension loadings properties, respectively. The result revealed that FA-SCFRC generally has enhanced properties compared to FA-SPC. In a nutshell, FA-SCFRC with a 10% fly ash content and 2% coconut fiber content showed better overall mechanical properties than fly ash plain concrete. Khan et al [29] reported on coconut fibers in concrete with different silica fume contents. The energy absorption and toughness indexes of silica-fume plain concrete (S-PC) and silica-fume coconut fiber reinforced concrete (S-CFRC) with the addition of 5%, 10%, 15%, and 20%, silica fume content by mass were taken in consideration. The length of coconut fiber was 50 mm and the content was 2% by mass. It was observed that S-CFRC has generally improved mechanical properties with a 2% coconut fiber and 15% silica fume content compared to the respective plain specimens.

### 1.4. Durability of Coconut Fiber Reinforced Concrete

Ramli et al. [30] studied the properties of CFRC in aggressive environments and reported that the incorporation of coconut fibers results in enhanced durability properties compared to PC. The strength and durability of concrete with coconut fibers under various aggressive environments like exposure to sea water and air, for various durations, were examined. Durability tests like carbonation depth, intrinsic permeability, and chloride penetration tests were carried out. The microstructure and mineralogy using scanning electron microscopy and X-ray diffraction was also studied. It was clearly observed from the obtained results that due to the addition of coconut fibers, both the compressive and flexural strength were improved by up to 13% and 9%, respectively. Apart from improved compressive and flexural properties, it was also reported that the durability properties, e.g., intrinsic permeability, carbonation depth, and chloride penetration were improved. It was noted from the microstructure and mineralogical studies that a seawater environment affected both the PC and CFRC samples. On other hand, it was mentioned that the use of a smaller amount of coconut fiber can be beneficial in terms of durability. Therefore, it was recommended that a lower amount of coconut fiber be used if durability is to be taken into consideration. By evaluating all the tested parameters, it was suggested that the approximate threshold value of coconut fiber is 1.2% (by binder volume), which would be suitable and beneficial for durability in long term, as well as for strength in all the tested aggressive environments.

### 1.5. Significance of Current Work

The disadvantages of HSC include brittleness, low tensile strength, and reduced resistance against crack propagation. The addition of fibers to composites has played an important role since biblical times. Brittle building materials like clay sun baked bricks were reinforced with straw, horse-hair, and other vegetable fibers [13,31,32]. The aim of this paper is to explain the present state of knowledge and technology concerning natural fiber reinforced concrete, and to discuss its applications. Lately, the use of natural fibers has been highlighted in many structural components. The benefits of natural fibers may also be considered from an environmental perspective. These fibers are inexpensive and are generally otherwise disregarded as waste [28,29]. Coconut fiber has the greatest toughness among all known natural fibers, which is the main reason for its selection in the current study. The research significance of this study is the exploration of the use of coconut fibers in high strength concrete with admixtures from a material properties perspective. Thus, it is vital to evaluate and predict the mechanical behavior of CFR-HSC for its possible use in civil engineering applications. Coconut fiber is very cheap and locally available in developing countries. The use of coconut fiber results in low-cost and more environmentally friendly concrete. Therefore, the optimal fiber length and content, especially when used in combination with silica fume and super plasticizer, need to be studies in detail. To the best of the authors’ knowledge, no work has reported on the optimization of coconut fiber length and content in HSC. In this study, coconut fibers of different lengths (25, 50, and 75 mm) and contents (0.5%, 1%, 1.5%, and 2% by mass) were incorporated into HSC to study its various mechanical properties. A silica fume content of 10% and super plasticizer content of 1% by mass were used. The mechanical properties of HSC were compared with those of coconut fiber reinforced high strength concrete (CFR-HSC). The compressive strength (σ), static modulus of elasticity (E_static_), energy absorption in compression, toughness index in compression (TIC), splitting tensile strength (STS), modulus of rupture (MOR), energy absorption in flexure, and toughness index in flexure (TIF) of the resulting concrete are discussed in detail.

## 2. Experimental Program

### 2.1. Material Properties

**Figure 1:**
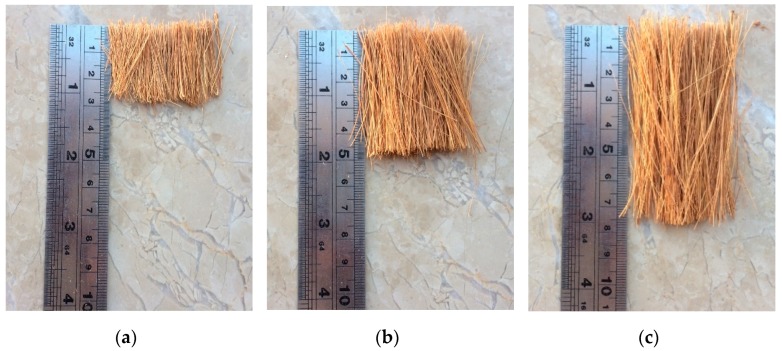
Coconut fibers used in experimental program with lengths (**a**) 25, (**b**) 50, and (**c**) 75 mm.

Type I Portland cement produced by Bestway (Islamabad, Pakistan) was used in the present study. Silica fume (Sika chemicals, Rawalpindi, Pakistan) and super-plasticizer (Sika Viscocrete 3110, Sika chemicals, Rawalpindi, Pakistan) were used. Locally available, coarse aggregate with 12.5 mm maximum size and sand with a fineness modulus (FM) of 2.7 were used. Both coarse aggregate and sand were washed and air dried before use. Coconut fibers with an average diameter of 0.32 mm were imported from Colombo, Sri Lanka. The coconut fibers with 25, 50, and 75 mm lengths (refer Figure 1) were soaked in water for 30 min to soften them before washing. Soaking and washing was performed three times to remove all the dust particles. The fibers were then dried in open air before use. A similar procedure for soaking and washing is also reported in the literature [22].

### 2.2. Mix Design and Casting Procedure

Table 2 shows the fiber lengths and contents for various concrete mixes. The mix quantities of cement, sand, aggregate, water, silica fume, and super plasticizer were 525, 785, 785, 184, 52.5, and 5.25 kg/m^3^, respectively. To mix all the ingredients, a concrete pan type mixer was used. To prepare the HSC, all the materials (cement, sand, aggregates, and silica fume) were put in the mixer pan. The super-plasticizer was mixed in water and added to the mixer. The mixer was rotated for three minutes. Ali et al. [22] reported the method for mixing coconut fibers in concrete to achieve a uniform dispersion of the fibers in the matrix and to avoid the balling effect. Silica fume was mixed with the cement. To prepare the CFR-HSC, a layer of coconut fibers was uniformly spread in the pan mixer; this first layer of fibers was hidden by spreading out the sand, aggregates, and cement. Then, another layer of fibers was uniformly spread over it followed again by a layer of sand, aggregate, and cement. This process was repeated until all the material was added in the pan mixer. Approximately three quarters of the total water containing super-plasticizer was added to the pan and the mixer was rotated for two minutes. Then, the remaining water was added to the mix and mixer was rotated for a further 3 min. The total mixing time for the CFR-HSC was five minutes.

To cast the specimens, the mix was poured into molds in three layers, and compaction of each layer was done with 25 blows of a tamping rod for both HSC and CFR-HSC. Since coconut fibers are flexible, there was no apparent damage to them due to tamping during casting. This observation was based upon a visual inspection of a few fibers which were removed from the concrete after tamping in the mold. All the specimens were demolded after 24 h and cured for 28 days before testing. Cylinders were casted to test the compressive and splitting-tensile strengths, while beams were casted for flexural strength tests. A set of three specimens was prepared for each test.

### 2.3. Testing Procedure

The slump test and density of the HSC was measured following the ASTM C143/143M-15a [33] and ASTM C138/C138M-13 [34] protocols, respectively. The same procedure was followed for CFR-HSC to determine its density because of the unavailability of ASTM standards for CFR-HSC. The density was defined as the ratio between the weight of the cylinder to its volume.

The ASTM C39/C39M-17b [35] and ASTM C496/C496M-17 [36] protocols were followed to test the compressive and splitting-tensile strengths of the cylinders for both the HSC and CFR-HSC. The maximum stress in the compressive stress-strain curve was taken as the compressive strength. The stress was calculated by load/area. The splitting-tensile strength was calculated according to the formula 2P/*π*LD. The peak load under the applied splitting-tensile load is notated as P, D is the diameter of the cylinder, and L is the length of cylinder. The ASTM C78/C78M-18 [37] and ASTM C1609/C1609M 12 [38] protocols were followed to perform flexural strength tests of the beams for both HSC and CFR-HSC. For scanning electron microscopy images, the VEGA3 TESCAN (Brno, Czech Republic) with a voltage of 10 kV was employed. The samples were coated with plasma before testing.

## 3. Results and Discussions

### 3.1. Slump of Fresh Concrete

**Figure 2:**
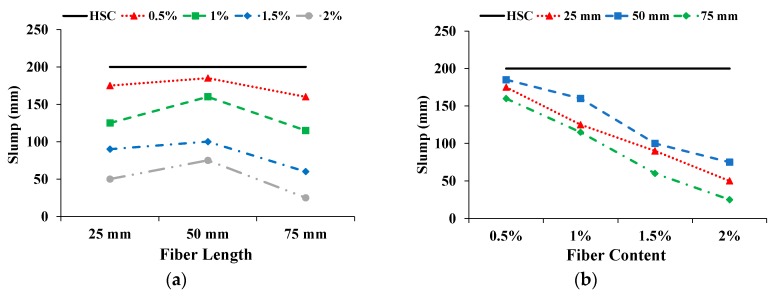
Influence on slump: (**a**) Fiber length; (**b**) Fiber content.

The effect on slump with increasing fiber length and content is shown in Figure 2. The solid straight line indicates the slump of the HSC. All CFR-HSCs showed decreased slump compared to HSC. With increasing the fiber length from 25 to 50 mm, the slump first improved and then deteriorated. The possible reasons are: (i) For 25 mm-long fibers, the fibers are more numerous, which decreases the workability of concrete; (ii) For 50 mm-long fibers, the fibers are less numerous, which results in increased slump, and (iii) For 75 mm-long fibers, the number of fibers further decreased, but longer fibers reduced the workability of concrete. With an increase in fiber content, slump is reduced. The higher quantity of fibers reduced the workability of the fresh concrete. The reduction in the slump of fresh concrete due to the addition of fibers was also reported by Khan and Ali [12], Ali et al. [22], and Iqbal et al. [39]. However, in spite of decreased slump, the CFR-HSC was found to be workable.

### 3.2. Density of Hardened Concrete

**Figure 3:**
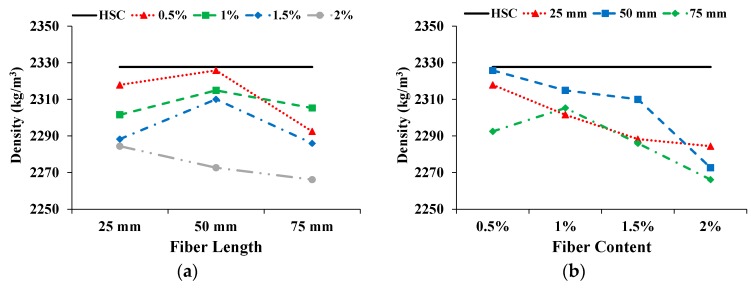
Influence on density: (**a**) Fiber length; (**b**) Fiber content.

As expected, the density of the CFR-HSC was less than that of HSC. The influence on density with increasing fiber length and content is shown in Figure 3. With the increase in fiber length, the density of CFR-HSC was enhanced and then reduced. With a further increase in fiber content, the density of the specimens decreased. As fibers are light, their addition to concrete creates voids in the matrix which decrease its density. The addition of low density coconut fibers results in a so-called filled void effect, which ultimately reduces the density compared to that of plain concrete. The decreased density with the incorporation of fibers is also reported in the literature [12,22]. The density of CFR-HSC with 75 mm fiber length and 2% content was reduced by 2.6% compared to HSC.

### 3.3. Compressive Properties

#### 3.3.1. Compressive Behavior

**Figure 4:**
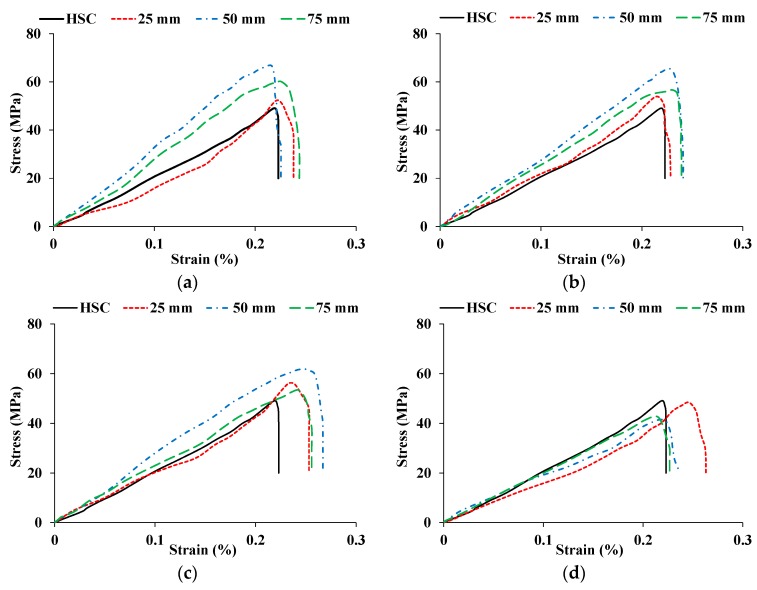
Stress-strain curves for HSC and CFR-HSC with (**a**) 0.5%, (**b**) 1%, (**c**) 1.5%, and (**d**) 2% fiber content.

**Figure 5:**
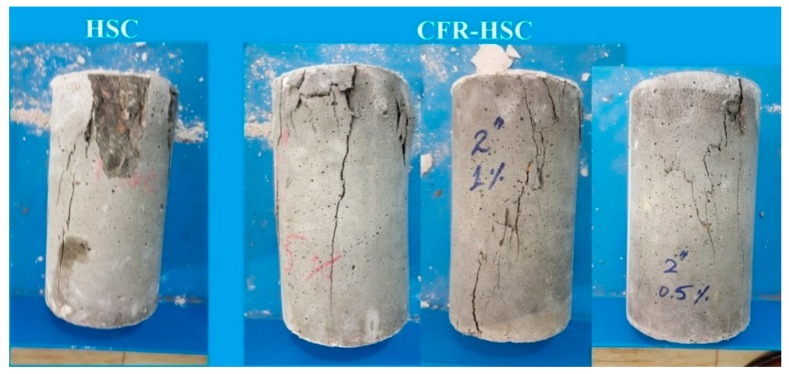
HSC and CFR-HSC specimens after compressive strength tests.

The stress-strain curves for HSC and CFR-HSC with the same fiber length but different content ratios are shown in Figure 4. As expected, CFR-HSC showed greater strength and strain values than HSC. Also, CFR-HSC showed substantially improved strain energy absorption after peak stress compared to HSC. With a 25 mm fiber length (Figure 4a), a little increase in peak stress was observed with all fiber contents, while a substantial increase in strain at peak stress was observed at higher fiber contents. At 50 mm fiber length (Figure 4b), a further increase in peak stress was observed at lower fiber contents, while maximum strain at peak stress was observed for 1.5% fiber content. For 75 mm fiber length (Figure 4c), the peak stress as well as strain at peak stress were reduced compared to 50 mm fiber length, but were higher than those of HSC and CFR-HSC with 25 mm fiber length. The HSC and CFR-HSC specimens after the compressive strength test are shown in Figure 5. At maximum load, concrete pieces from HSC were chipped off, while for CFR-HSC, pieces of concrete were held together. The reason for this is the presence of fibers which provided a bridging effect in the CFR-HSC. Also, it was visually observed that crack width and length, and the number of cracks, were greater in HSC than in CFR-HSC.

#### 3.3.2. Static Modulus of Elasticity (E_static_)

**Figure 6:**
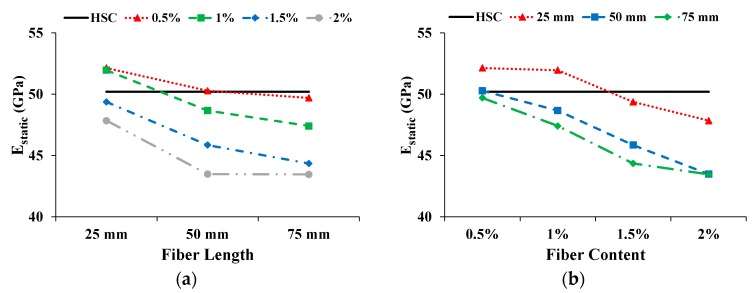
Influence on E_static_: (**a**) Fiber length; (**b**) Fiber content.

Figure 6 shows the effect on E_static_ with increasing fiber length and content. The solid straight line shows the E_static_ of HSC. The E_static_ of CFR-HSC is reduced with the increase in fiber length and content. A similar trend was also reported by Ali et al. [22]. Only the CFR-HSC with 25 mm fiber length and 0.5% and 1% content showed enhanced E_static_ compared to HSC. All the other CFR-HSCs showed less E_static_ than HSC. The E_static_ of CFR-HSC with 50 mm and 75 mm fiber lengths and 2% content was reduced by 13.4% compared to HSC. A reduction in E_static_ due to the addition of fibers in concrete is also reported in the literature [22].

#### 3.3.3. Compressive Strength (σ)

**Figure 7:**
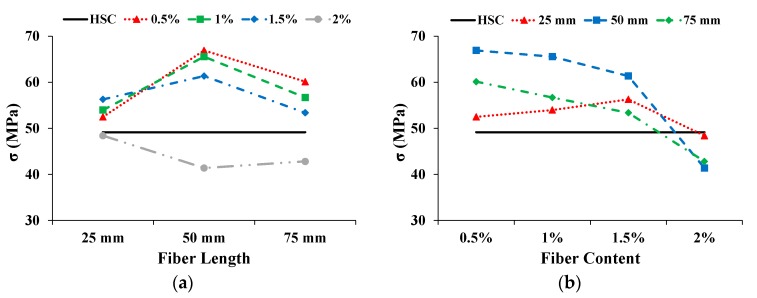
Influence on σ: (**a**) Fiber length; (**b**) Fiber content.

The influence on σ with increasing fiber length and content is shown in Figure 7. The solid straight line shows the σ of the HSC. The σ of CFR-HSC first increased and then reduced with increasing fiber length. For the 25 mm long fiber, the σ was enhanced with an increase in fiber content up to 1.5% and then decreased when the fiber content was increased to 2%. However, for 50 mm and 75 mm long fibers, the σ is decreased with the increase in fiber content. The reduction in σ may be due to: (i) the workability of fresh concrete decreasing due to the higher content and longer length of fibers, and because proper compaction was not done during the casting of the specimens, resulting in the creation of air voids; or (ii) the dilution of the cement matrix/hardened cement paste due to the addition of fibers. The improvement in σ due to the addition of fibers is also reported by Afroughsabet and Ozbakkaloglu [1], Ali et al. [22], and Ramli et al. [30].

#### 3.3.4. Energy Absorption in Compression and Toughness index

**Figure 8:**
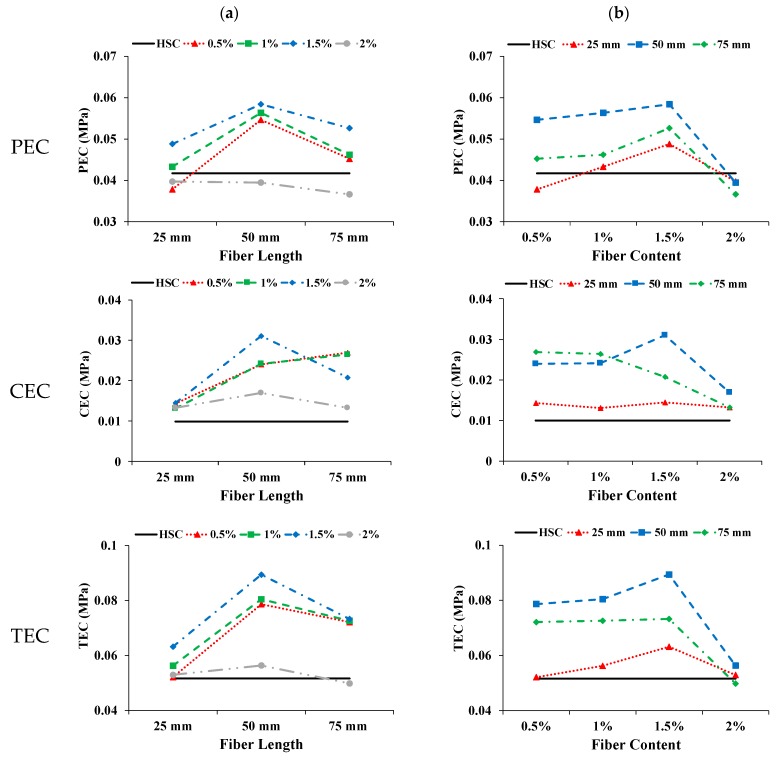

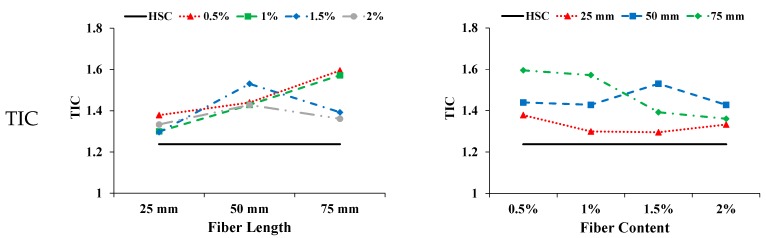
Influence of coconut fiber on PEC, CEC, TEC, and TIC: (**a**) Length; (**b**) Content.

The areas below the stress–strain curve up to the first crack, from the stress of the first crack up to the ultimate stress, and from zero to the ultimate stress, are taken as the pre cracked energy absorption in compression (PEC), cracked energy absorption in compression (CEC), and total energy absorption in compression (TEC), respectively. The ratio of TEC to PEC was calculated as the toughness index in compression (TIC). Figure 8 shows the influence of coconut fiber on different parameters with increasing fiber length and content. The CFR-HSC showed increased PEC compared to HSC. The PEC of CFR-HSC first improved and then deteriorated with increasing fiber length. With increasing fiber content up to 1.5%, the PEC was enhanced and then deteriorated. The PEC of CFR-HSC with 50 mm fiber length and 1.5% content had the maximum value, i.e., 40% higher than that of HSC. As expected, the CEC of CFR-HSC was higher than that of HSC. The addition of fibers provided significant stress resistance after the first crack by bridging the cracks, resulting in higher CEC. The CEC of CFR-HSC with 0.5% and 1% fiber content increased with increasing fiber length. In contrast, the CEC of CFR-HSC with 1.5% and 2% fiber content first improved and then deteriorated with increasing fiber length. The CEC of CFR-HSC reduced with increasing fiber content.

The possible reason for this may be improper compaction due to less workability of the fresh mix and the creation of air voids in the matrix because of higher fiber content, resulting in lower energy absorption after the first crack occurred. The CFR-HSC with 50 mm fiber length and 1.5% content had the highest CEC value. The solid straight line is the TEC of HSC. With increasing fiber length, the TEC of CFR-HSC was first enhanced and then deteriorated. For each fiber content, 50 mm long fibers had the maximum TEC value. The possible reasons for this are: (i) at 25 mm fiber length, the fibers are more for bridging the cracks, but their embedment length is short, resulting in fiber pull-out; (ii) when the fiber length is 50 mm, relatively fewer fibers are present, but the embedment length is sufficient to hold the cracks together, resulting in a higher TEC value; (iii) when the fiber length is 75 mm, the embedment length is improved the presence of fewer fibers results in fiber breaking and a reduced TEC value. The TEC of CFR-HSC increases with increasing fiber content up to 1.5%, and then deteriorates. The possible reasons for this may be improper compaction due to less workability of the fresh mix, and cement paste dilution at higher fiber contents and longer lengths. The TEC of CFR-HSC with 50 mm fiber length and 1.5% content was improved by 73.2% compared to HSC. The TIC of CFR-HSC was always higher than that of HSC, as the incorporation of fibers provided stress resistance, especially after the maximum load by bridging across the cracks. The energy absorption capacity of concrete and toughness index, in compression, was improved by the addition of fibers, as also reported by Ali et al. [22], Zia and Ali [40], and Khan and Ali [41].

### 3.4. Splitting-tensile Properties

**Figure 9:**
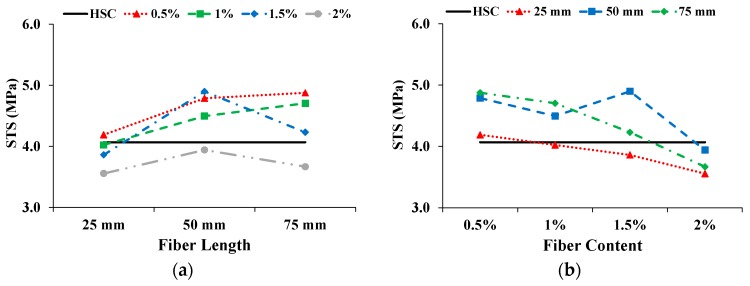
Influence on STS: (**a**) Fiber length; (**b**) Fiber content.

**Figure 10:**
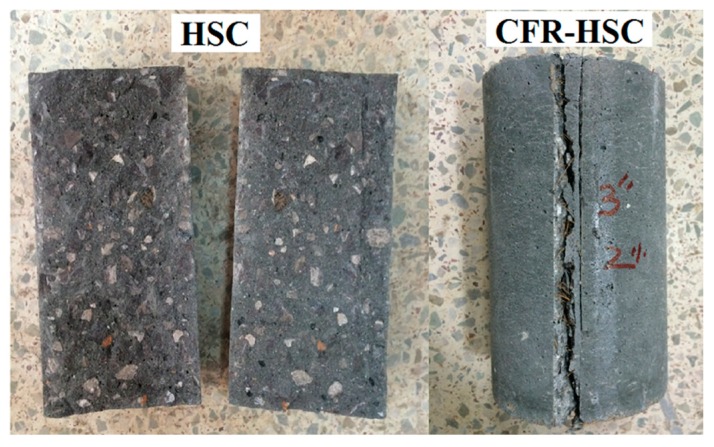
HSC and CFR-HSC specimens after STS test.

The STS was calculated from the maximum load taken from the splitting-tensile load-time curve. Figure 9 shows the effect on STS with increasing fiber length and content. The solid straight line shows the STS of HSC. With increasing fiber length, the STS of the CFR-HSC with 0.5% and 1% fiber contents kept increasing, while at 1.5% and 2%, the STS was first enhanced and then slightly deteriorated. The STS is reduced with increasing fiber content. However, in the case of 50 mm fiber length, the STS has a maximum value at 1.5% fiber content, which is 20.4% more than that of HSC. For shorter fiber lengths, the STS of CFR-HSC was less than that of HSC because of insufficient embedment length to bridge the cracks. For longer fibers, the sufficient embedment length was available for bridging the cracks, resulting in increased STS compared to HSC. At higher fiber contents, the STS of CFR-HSC was reduced because of the creation of voids in the matrix and improper compaction due to higher fiber contents, which resulted in less workability. A similar trend in CFRC is also described by Ali et al. [22]. The improvement in STS due to the addition of fibers in concrete is also reported in the literature [1,12,39,41]. The presence of fibers in CFR-HSC results in a bridging effect that holds the two pieces together. The samples of HSC and CFR-HSC after testing are shown in Figure 10. The CFR-HSC samples were intentionally separated after testing to observe the fiber failure. It was visually observed that some fibers were pulled-out, and that most were broken at the fracture surface. The embedment length of the fibers in the concrete increased with increasing fiber length, resulting in a reduced amount of fiber pull-out.

### 3.5. Flexural Properties

#### 3.5.1. Flexural Behavior

**Figure 11:**
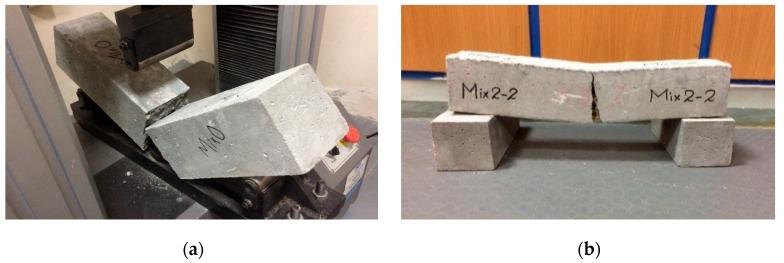

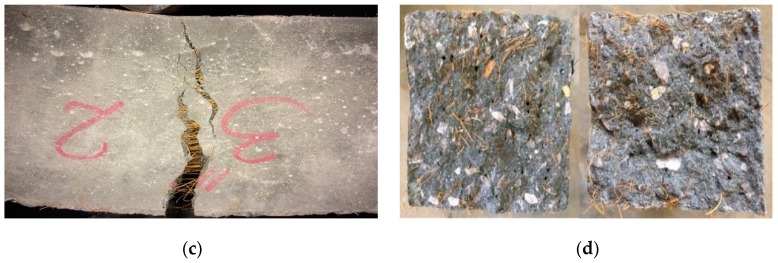
Beams test: (**a**) Tested HSC beam; (**b**) Tested CFR-HSC beam; (**c**) Fiber bridging in beam; (**d**) Cross-section of tested CFR-HSC beam.

**Figure 12:**
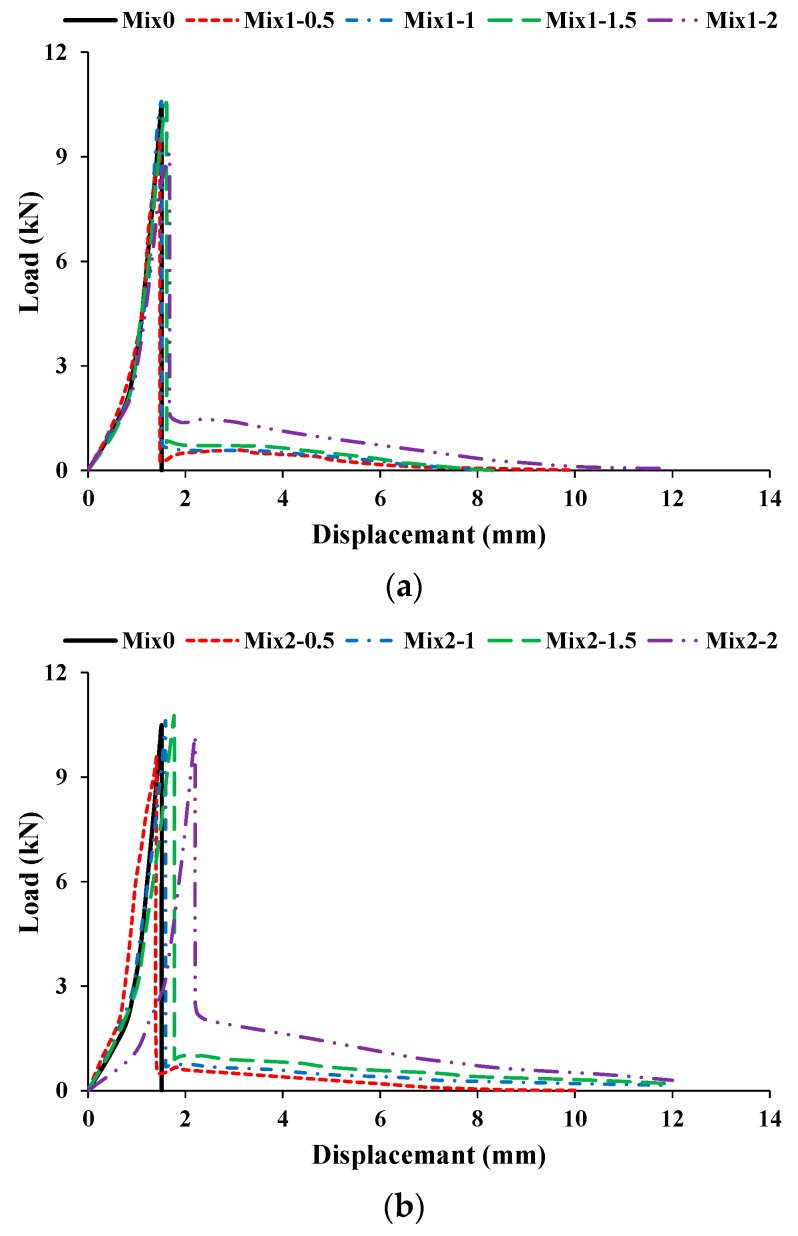

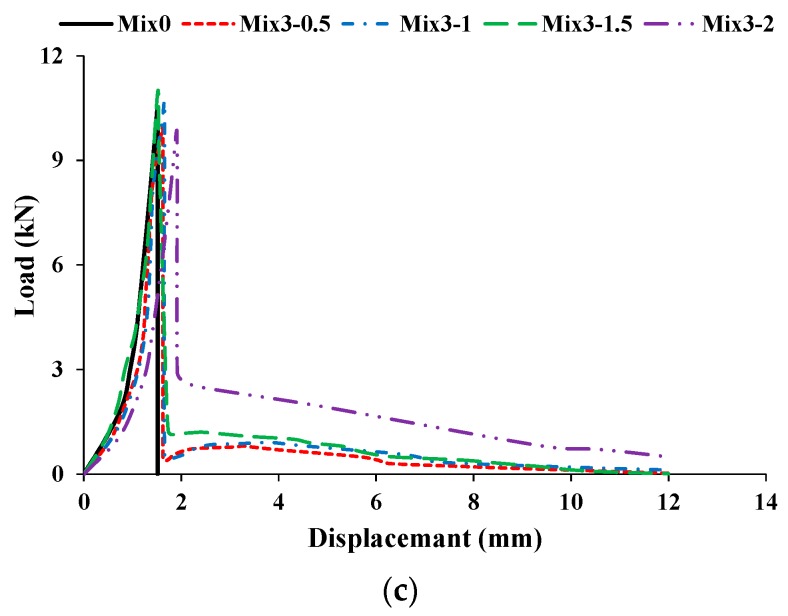
Load-displacement curves for HSC and CFR-HSC beams with (**a**) 25, (**b**) 50, and (**c**) 75 mm long fibers.

During the flexural-strength testing of beams, flexural load-displacement curves were recorded for all specimens. The HSC beams suddenly fragmented into two pieces at peak load (Figure 11a). However, CFR-HSC were held together even after the peak load (Figure 11b–d). This is due to the bridging effect of the fibers in the CFR-HSC beams, which held the beam together after cracking. The flexural load-displacement curves for HSC and CFR-HSC are shown in Figure 12. To observe the fiber failure mode in CFR-HSC, the beams were intentionally broken into pieces. The fiber failures were of two types, fiber pull-out and fiber breakage. The fiber pull-out failure was reduced with increasing fiber length. The reason for this was that for shorter fiber lengths, the embedment length of the fiber in the concrete was shorter, resulting in increased pull-out failure; whereas for longer fibers, embedment length increases, thereby resisting the pull-out of fibers and resulting in increased fiber breakage failure.

#### 3.5.2. Modulus of Rupture (MOR)

**Figure 13:**
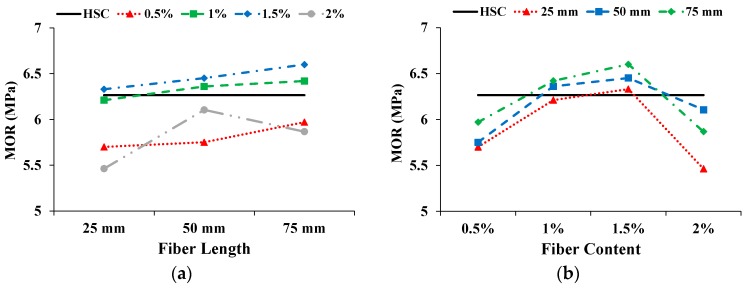
Influence on MOR: (**a**) Fiber length; (**b**) Fiber content.

The MOR was calculated from the peak load in the load-displacement curve. Figure 13 shows the effect on MOR with increasing fiber length and content. The solid straight line indicates the MOR of HSC. The MOR was enhanced with increasing coconut fiber length; a similar trend was also reported by Ali. et al [22]. Only with a 2% fiber content, the MOR of CFR-HSC first improved and then deteriorated with increasing fiber length. With increasing fiber content up to 1.5%, the MOR was greater, while a low MOR was observed upon further increasing the fiber content. Compared to HSC, the MOR of CFR-HSC with a 1.5% fiber content and 75 mm length was enhanced by 5.4%. The MOR of CFR-HSC with a 0.5% fiber content was less than that of HSC. The reason for this is that at a 0.5% fiber content, the fibers are fewer, and unable to resist flexural load. The MORs of CFR-HSC with 1% and 1.5% fiber contents were higher than that of HSC because there was a sufficient amount of fibers to resist flexural load. With a 2% fiber content of CFR-HSC, the MOR was again less than that of HSC. The possible reasons for this may be: (i) the workability of fresh concrete decreased due to a higher content and longer length of fibers, and because proper compaction was not done during the casting of the specimens, resulting in the creation of air voids; (ii) the dilution of cement matrix/hardened cement paste due to the addition of fibers. The addition of fibers in concrete resulted in enhanced MOR; this finding is consistent with the results of Afroughsabet and Ozbakkaloglu [1], and Iqbal et al. [39].

#### 3.5.3. Energy Absorption in Flexure and Toughness Index

**Figure 14:**
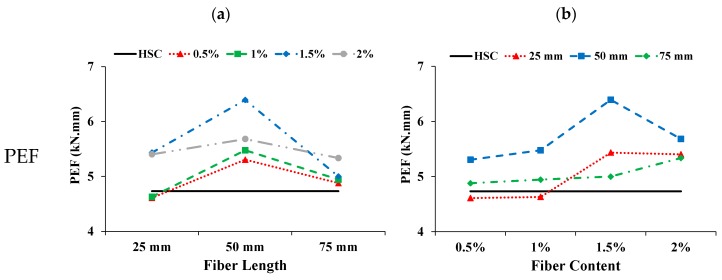

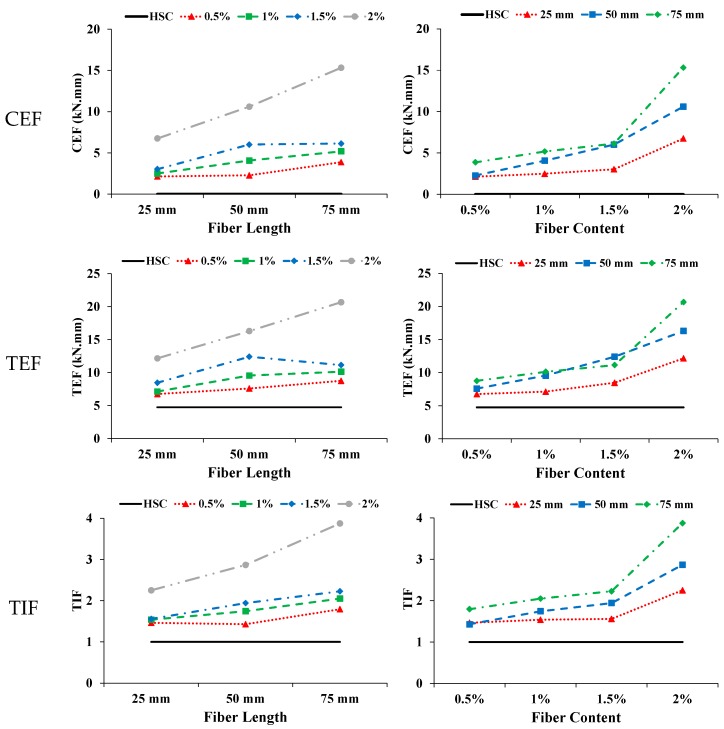
Influence of coconut fiber on PEF, CEF, TEF, and TIF: (**a**) Length; (**b**) Content.

The pre cracked energy absorption in flexure (PEF) is taken as the area below the load-displacement curve up to peak load. The cracked energy absorption in flexure (CEF) is taken as the area below the load–displacement curve from peak load up to the ultimate load. The ultimate load is taken as the load at which test is stopped. The total area below the load-displacement curve from zero to the ultimate load is taken as the total energy absorption in flexure (TEF). The toughness index in flexure (TIF) is calculated as the ratio of total energy absorption in flexure to the pre cracked energy absorption in flexure (i.e., TEF/PEF). Figure 14 present the influence on PEF with increasing fiber length and content. The PEF of CFR-HSC was mostly higher than that of HSC. The PEF is improved with the addition of coconut fibers up to the optimum content; beyond the optimum content, the PEF decreases with increasing fiber length. Larger fibers result in reduced workability which ultimately decrease the PEF. The PEF was enhanced with increasing fiber content up to 1.5%, and then deteriorated on further increasing the fiber content. The reasons for this may be improper compaction at the time of casting because of less workability and cement dilution with a higher fiber content. The effect on CEF with increasing fiber length and content is shown in Figure 14. The CEF of HSC is zero, as it was suddenly broken into two halves at peak load. The CEF of CFR-HSC improved with increasing fiber length and content. The possible reasons for this are: (i) when the fiber length is short, the embedment length of fibers in concrete is shorter, resulting in pull-out failure and lower CEF; (ii) with longer fibers, the embedment length of fibers in concrete increases, making them able to resist significant flexure load before failure, ultimately showing an increased CEF value; (iii) with increasing fiber content, more fibers are available to bridge the cracks, resulting in an increased value of CEF. The TEF of HSC is less than that of CFR-HSC. The TEF of CFR-HSC is enhanced with increasing fiber length and content. The TEF of CFR-HSC with a 1.5% fiber content was first improved and then slightly deteriorated with increasing fiber length. The CFR-HSC with a 2% content and 75 mm length had the maximum TEF value. The TIF of CFR-HSC was higher than that of HSC. The TIF of CFR-HSC increased with increasing fiber length and content. The possible reasons for this are: (i) with increasing fiber content, more fibers are available for bridging cracks, which increases the TEF, thus increasing the TIF; (ii) with increasing fiber length, the embedment length of fibers increases, resulting in increased TIF. The energy absorption capacity and toughness index of concrete in flexure is increased because of the incorporation of fibers, as also reported by Khan and Ali [12], and Zia and Ali [40].

### 3.6. Microstructure Study

**Figure 15:**
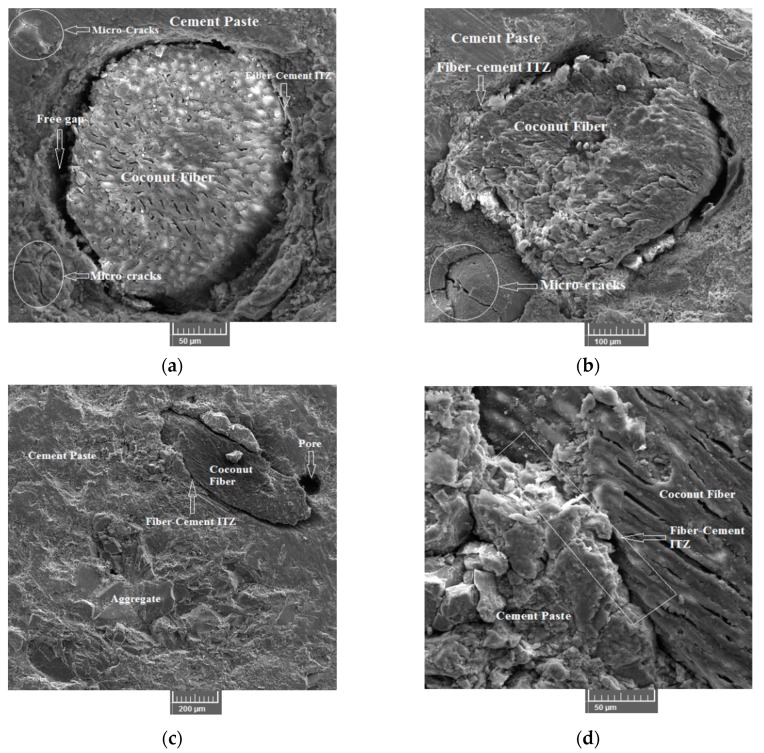

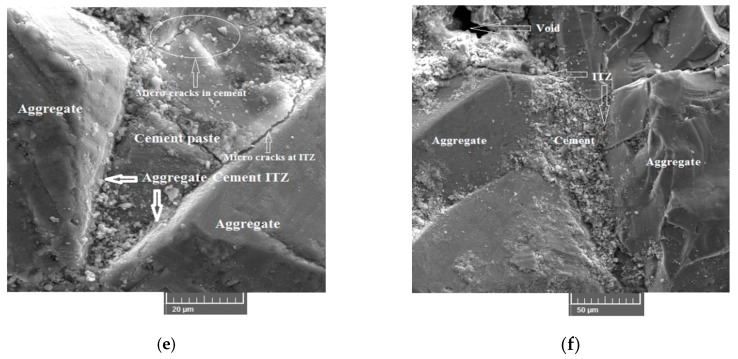
Microstructure of coconut fiber reinforced high strength concrete: (**a**) Gap in fiber-cement paste ITZ and micro-cracks; (**b**) Micro-cracks and fiber-cement paste ITZ; (**c**) Pores in the matrix; (**d**) Fiber-cement paste ITZ; (**e**) Micro-cracks in cement paste and aggregate-cement paste ITZ; (**f**) Aggregate-cement paste ITZ.

A SEM analysis was performed to study the microstructure of the CFR-HSC. The samples were prepared from CFR-HSC beams after performing flexural strength tests. The objective of the SEM analysis was to study the fiber-cement paste and aggregate-cement paste interfacial transition zones (ITZs), micro-cracks, and their propagation in the matrix. A free gap was observed at the ITZ between the fiber and cement paste (Figure 15a,b), which indicates a fiber debonding failure. This is because CFR-HSC samples have reduced MOR compared to HSC. However, from Figure 15c,d, it can be seen that a proper bond between the fiber and cement paste occurred, i.e., there was no gap, resulting in increased mechanical properties. It can be also concluded that slippage of fiber in the matrix is the main reason for the higher energy absorption. Figure 15a,b,e shows the micro-cracks in the cement paste and aggregates. Crack propagation was observed through the aggregate and cement paste, which indicates a strong fiber-cement paste ITZ and improved microstructure. The micro-structure of the matrix improved with the addition of silica-fume due to its high pozzolanic activity. Pozzolanic activity means that during hydration under an alkaline environment, C–S–H gel is formed by silica-fume reacting with Ca(OH)_2_. The C–S–H gel reduces the porosity of the matrix by filling the pores, which ultimately enhances the matrix strength. The silica-fume cannot hydrate directly with water, and hence, reacts with Ca(OH)_2_. Also, due to the orientation and crystallinity of Ca(OH)_2_, an adverse effect on the interfacial and microstructure properties can occur. The strength of the matrix was enhanced by the addition of silica-fume to a certain extent, but the addition of fibers in higher quantities may result in low bond strengths due to heterogeneity in the mix. Also, due to low bond strength, fiber debonding can occur. The inclusion of coconut fiber in the matrix led to new interfaces within the matrix, resulting in weak links between the coconut fiber and the matrix. Due to the debonding of the coconut fiber from the matrix, free gaps occurred (refer Figure 15a). The major reason for this is the addition of coconut fiber in higher quantities, which reduces the bond strength of the matrix due to the higher water:cement ratio. This free gap may also occur due to fiber fractures. This free gap resulted in reduced toughness and post cracking performance of the matrix. A good bond between coconut fibers and the cement matrix should have no gaps, resulting in enhanced mechanical properties, as shown in Figure 15d. Also, it is important to note that the slippage of fibers with respect to the matrix is the main reason for the higher energy dissipation and toughness. Within the matrix, the fibers are surrounded by cement paste, which improves the matrix strength due to a stress transfer mechanism between the matrix and reinforced fibers. Moreover, to some extent, tensile stresses generated by applying a load are also resisted by fibers, which helps to retain the compact micro-structure by resisting crack propagation.

## 4. Discussion

In this study, coconut fibers having lengths 25, 50, and 75 mm and 0.5%, 1%, 1.5%, and 2% content by mass, are incorporated in HSC having mix design ratio of 1:1.5:1.5 for cement, sand and aggregate, respectively. The w:c ratio is kept at 0.35 for both HSC and CFR-HSC. The silica fume content of 10%, by cement mass, are used in all concrete mixes for achieving high strength of concretes. However, the super-plasticizer content of 1% by mass, are added to all CFR-HSCs for improving the workability of fresh mix. The incorporation of coconut fibers in HSC increased the σ, E_static_, STS, MOR as well as improved post cracking behavior in compression, splitting-tensile and flexure. With optimized CFR-HSC (Mix2-1.5), the σ, PEC, CEC, TEC, and TIC is increased by 24.8%, 40%, 213%, 73.1%, and 23.6%, respectively, compared to that of HSC. However, with the same CFR-HSC, the E_static_ is decreased by 8.7%, than that of HSC. The possible reasons for the improved properties of CFR-HSC than that of HSC are: (i) Fibers restrained crack extension and delayed cracks growth rate which results in increased σ of CFR-HSC; (ii) Incorporation of coconut fibers results in elastic behavior thus reducing E_static_; (iii) The improved energy absorption of CFR-HSC is due to fiber bridging effect and better post cracking behavior. With optimized CFR-HSC (Mix2-1.5), the STS is increased by 20.4%, compared to that of HSC. The reasons for improved splitting-tensile properties of CFR-HSC are improved microstructure and fiber bridging effect. With optimized CFR-HSC (Mix2-1.5), the MOR, PEF, TEF, and TIF is increased by 3%, 35.1%, 162%, and 94%, respectively, compared to that of HSC. The HSC beam suddenly broken in to two halves at peak load during flexural strength test, thus giving no CEF value; while all CFR-HSC showed significant CEF values resulting in enhanced TEF. The reasons are already explained in Section 3.5.3.

Table 3 summarizes all the mechanical properties of the HSC and CFR-HSC. Overall, the best properties were obtained from CFR-HSC with 50 mm-long fibers and 1.5% fiber content by mass. The σ, TEC, STS, TES, MOR, and TEF of CFR-HSC with 50 mm long fibers and 1.5% content were improved by 24.8%, 71.2%, 20.4%, 120%, 3%, and 162%, respectively, compared to HSC. In contrast, for the same CFR-HSC, the slump and E_static_ were reduced by 50% and 8%, respectively. The ACI committee 234 [42] reported that the use of silica fume as a replacement of cement in concrete decreases permeability and increases durability and resistance to chemical attack. Thus, concrete with silica fume has better performance in structural applications. Also, in addition to silica fume, the incorporation of coconut fibers would lead to better durability properties and resistance to deleterious effects in aggressive environments, as also reported by Ramli et al. [30]. It has been suggested that the approximate threshold value of coconut fiber is 1.2%, which would be suitable and beneficial for durability in the long term, as well as for strength in all of the tested aggressive environments. In this study, the optimum threshold for CFR-HSC was shown to be 50 mm long fibers and a 1.5% fiber content, which is closer to the optimal fiber content reported in the literature for better durability. The improved strengths and better post cracking behavior favor the use of CFR-HSC in structural applications.

## 5. Conclusions

In this study, coconut fibers of different lengths (25, 50, and 75 mm) and contents (0.5%, 1%, 1.5% and 2% by mass) were added to high strength concrete (HSC) to investigate its mechanical properties for use in structural applications. The results for the coconut fiber reinforced high strength concrete (CFR-HSC) were compared with those of HSC with the same mix design. The following conclusions were made:

- The slump and density of CFR-HSC were reduced compared to those of HSC. With changing fiber length and content, the slump of CFR-HSC was reduced up to 87.5%, and the density is reduced up to 2.7% compared to HSC.
- The compressive strength (σ) of CFR-HSC was increased with lower fiber content, while σ was decreased with the incorporation of a higher fiber content compared to HSC. The σ of CFR-HSC with 50 mm-long fibers and 1.5% fiber content was enhanced by 25% compared to that of HSC.
- There was an increase of 20.4% and 3% in splitting-tensile and flexural strengths, respectively, for CFR-HSC with 50 mm long fibers and 1.5% content compared to those of HSC.
- In comparison to HSC, the total energy absorption in the compression and flexure of CFR-HSC with 50 mm-long fibers and 1.5% fiber content were improved by 72.5% and 162%, respectively. In addition, the toughness index in compression and flexure for the same CFR-HSC increased by 23.4% and 94%, respectively, compared to HSC.
- The best overall results for CFR-HSC were observed with the addition of 50 mm long coconut fibers and with a 1.5% fiber content.

Based on above results, the improved mechanical properties of CFR-HSC with 50 mm long coconut fibers and a 1.5% fiber content favor its use in concrete structural applications. However, the durability properties of CFR-HSC need to be evaluated in future due to the organic nature of coconut fiber.

## Figures and Tables

**Table 1:** Comparison of PC and CFRC mechanical properties according to Baruah and Talukdar [24].

Fiber Volume Fraction (%)	Compressive Strength (MPa)	Split Tensile Strength (MPa)	Modulus of Rupture (MPa)	Toughness Index (I_5_)
-	21.42	2.88	3.25	1.934
0.5	21.70	3.02	3.38	2.165
1.0	22.74	3.18	3.68	2.109
1.5	25.10	3.37	4.07	2.706
2.0	24.35	3.54	4.16	2.345

**Table 2:** Mix proportions of fiber lengths and contents for concrete mixes.

Concrete Type	Mixture ID	Fiber Length (mm)	Fiber Content (%)
HSC	Mix0	-	-
CFR-HSC	Mix1-0.5	25	0.5
	Mix1-1	25	1
	Mix1-1.5	25	1.5
	Mix1-2	25	2
	Mix2-0.5	50	0.5
	Mix2-1	50	1
	Mix2-1.5	50	1.5
	Mix2-2	50	2
	Mix3-0.5	75	0.5
	Mix3-1	75	1
	Mix3-1.5	75	1.5
	Mix3-2	75	2

**Table 3:** Mechanical properties of CFR-HSC.

Concrete Type	E_static_ (GPa)	σ (MPa)	TEC (MPa)	STS (MPa)	MOR (MPa)	TEF (kN.mm)	Density (kg/m^3^)
**HSC**	50.21	49.14	0.052	4.07	6.26	4.73	2328
**CFR-HSC with maximum values**	52.14 (0.5%, 25 mm)	66.9 (0.5%, 50 mm)	0.089 (1.5%, 50 mm)	4.90 (1.5%, 50 mm)	6.60 (1.5%, 75 mm)	20.66 (2%, 75 mm)	2326 (0.5%, 50 mm)
**CFR-HSC with minimum values**	43.46 (2%, 75 mm)	41.36 (2%, 50 mm)	0.050 (2%, 75 mm)	3.56 (2%, 25 mm)	5.46 (2%, 25 mm)	6.74 (0.5%, 50 mm)	2266 (2%, 75 mm)
**Recommended CFR-HSC (1.5% fiber content, 50 mm-long fibers)**	45.85	61.34	0.089	4.9	6.45	12.4	2310
